# Prion protein signaling as therapeutic target in human tumors

**DOI:** 10.1186/1753-6561-7-S2-K23

**Published:** 2013-04-04

**Authors:** Vilma Regina Martins

**Affiliations:** 1 International Center of Research and Training (CIPE), Hospital A.C. Camargo, São Paulo, SP, Brazil

## 

Prions are proteinaceus infectious agents associated to invariable fatal neurodegenerative disorders named transmissible spongiform encephalopathies or prion diseases. The mechanism associated with disease propagation lays on post-translation modifications of the cellular prion protein (PrP^C^), which acquires altered conformation, form aggregates and is resistant to proteolysis. PrP^C^ is a cell surface glycosyphosphatidylinositol (GPI)-anchored protein of 208-209 aminoacids which is codified by a single exon and whose expression is developmentally regulated. In adult animals PrP^C^ is highly expressed in the central nervous system but peripheral nervous system and other organs or tissues also express this protein.

Initial experiments conducted with PrP^C^-null mice established that this protein is absolutely necessary to propagate prion infection. Remarkably, these animals had no gross anatomical abnormalities or behavior alterations although higher neuronal sensitivity to stress conditions such us seizures and hypoxic-ischemic injury are present. These data pointed that PrP^C^ would be unnecessary for embryonic development or some compensatory mechanism might be present in these animals. In fact, the later hypothesis was supported by findings that demonstrated that deletion of specific PrP^C^ domains cause severe neurodegeneration in mice.

The use of cellular models led to the characterization of PrP^C^ ligands and related signaling pathways allowing the definition of PrP^C^ cellular functions. We identified three major ligands for PrP^C^, two proteins of the extracellular matrix, laminin and vitronectin, and a secreted co-chaperone the stress inducible protein 1 (STI1) or Hop (its human homologue). Comparative experiments using neurons derived from wild type and PrP^C^-null mice demonstrated that PrP^C^ binding to vitronectin promoted axonal growth in dorsal root ganglion neurons (DRG). In addition, PrP^C^ interaction with laminin activated metabotropic glutamate receptors, Ca^2+^ mobilization and PKC activation promoting neuronal plasticity and memory formation in rats. STI1 was the most explored PrP^C^ ligand. The PrP^C^-STI1 engagement induces PKA an ERK1/2 activation, which increase neuronal survival and differentiation. The signaling pathways modulated by PrP^C^-STI1 also included Ca^2+^ influx by activation of α-7 nicotinic acetylcholine receptor, induction of PI3K and mTOR. These last two pathways were associated to increased protein synthesis. An increment on the self-renewed of neuronal progenitors is also modulated by PrP^C^-STI1 engagement.

The co-chaperone STI1 is known to interact with PrP^C^ at the neuronal membrane and recently we demonstrated that this protein is abundantly secreted by astrocytes. Secreted STI1 plays an autocrine activity upon astrocytes modulating their proliferation and differentiation. The mechanism associated with STI1 secretion is under evaluation but preliminary results indicated that this protein is secreted by microvesicles derived from multivesicular bodies.

The involvement of PrP^C^ and STI1/Hop in cellular survival and differentiation raised questions regarding their involvement in tumoral processes. In fact, the literature has been pointed that PrP^C^ expression contributes to cancer progression and resistance to various cancer therapies. STI1/Hop expression was also associated with the proliferation of tumor cells. Our data pointed that STI1 secreted by glioblastoma cells has an autocrine function mediating proliferation of these cells by binding to PrP^C^. We are presently evaluating how to interfere with the PrP^C^-STI1 binding in order to modulate tumor growth.

Together our results point that PrP^C^-STI1 engagement is an interesting therapeutic target for both neurodegeneration and cancer and investments should be done to address these issues.

## Competing interests

There are no competing interests in this presentation.

